# Correction: Mathematical modelling and control of African animal trypanosomosis with interacting populations in West Africa—Could biting flies be important in maintaining the disease endemicity?

**DOI:** 10.1371/journal.pone.0253677

**Published:** 2021-06-17

**Authors:** Paul Olalekan Odeniran, Akindele Akano Onifade, Ewan Thomas MacLeod, Isaiah Oluwafemi Ademola, Simon Alderton, Susan Christina Welburn

The word "maintaining" is misspelled in the article title. The correct title is: Mathematical modelling and control of African animal trypanosomosis with interacting populations in West Africa—Could biting flies be important in maintaining the disease endemicity? The correct citation is: Odeniran PO, Onifade AA, MacLeod ET, Ademola IO, Alderton S, Welburn SC (2020) Mathematical modelling and control of African animal trypanosomosis with interacting populations in West Africa—Could biting flies be important in maintaining the disease endemicity? PLoS ONE 15(11): e0242435. https://doi.org/10.1371/journal.pone.0242435

## References

[1] Odeniran PO, Onifade AA, MacLeod ET, Ademola IO, Alderton S, Welburn SC (2020) Mathematical modelling and control of African animal trypanosomosis with interacting populations in West Africa—Could biting flies be important in main taining the disease endemicity? PLoS ONE 15(11): e0242435. 10.1371/journal.pone.0242435 33216770 PMC7679153

