# Supplementary material for: Mathematical modelling and control of African animal trypanosomosis with interacting populations in West Africa—Could biting flies be important in main taining the disease endemicity?
Source: PLoS One. 2020 Nov 20;15(11):e0242435. doi: 10.1371/journal.pone.0242435 (PMC7679153; doi:10.1371/journal.pone.0242435)
Supplement: S2 Fig — (PDF) [file pone.0242435.s002.pdf]

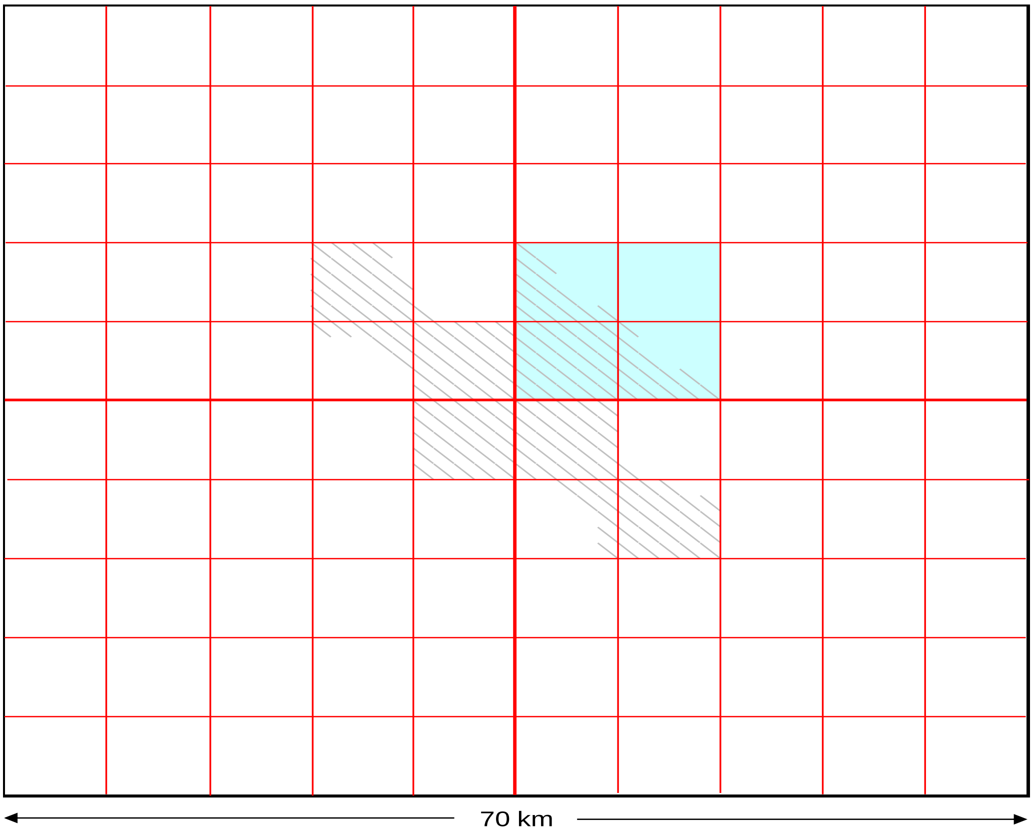

Map consists of 50 x 50 (= 2500) cells, each 1400 x 1400 m square

Adversity code:

Indicates stress due to factors other than baits to be deployed by the project

0. Nil
1. Low
2. Medium
3. High
4. Very high

Area to be baited
