# Supplementary material for: Mathematical modelling and control of African animal trypanosomosis with interacting populations in West Africa—Could biting flies be important in main taining the disease endemicity?
Source: PLoS One. 2020 Nov 20;15(11):e0242435. doi: 10.1371/journal.pone.0242435 (PMC7679153; doi:10.1371/journal.pone.0242435)
Supplement: S3 Fig — (PDF) [file pone.0242435.s003.pdf]

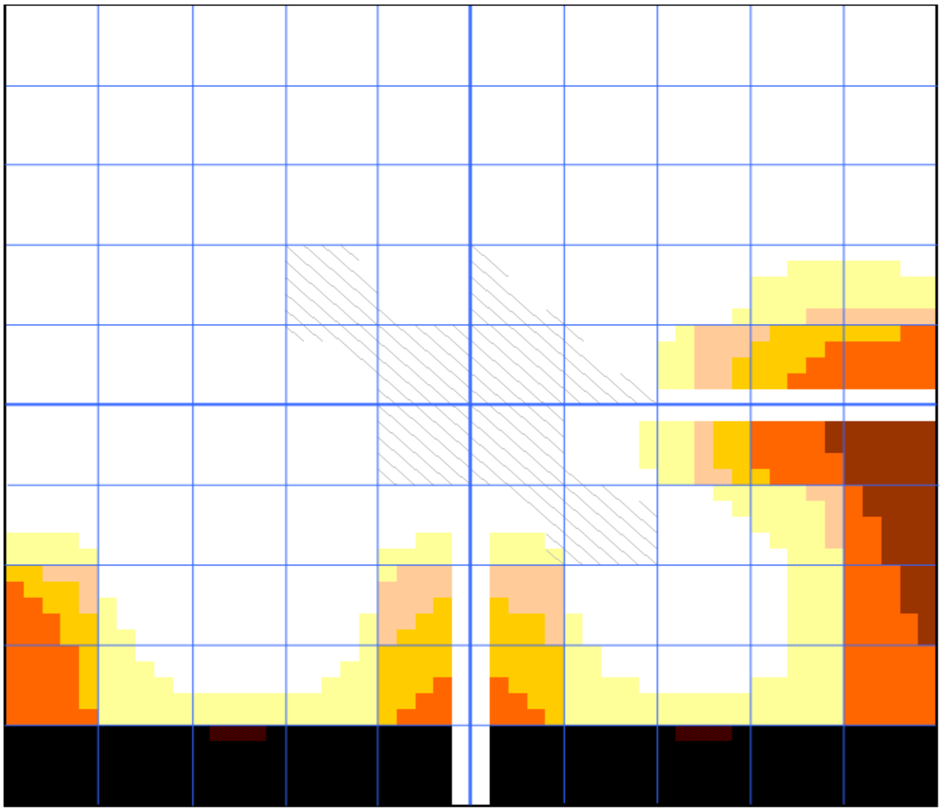

70 km

Map consists of 50 x 50 (= 2500) cells, each 1400 x 1400 m square

Tsetse species: *Riverine sp.*

Location in relation to the climatic limits of the species: 'Well inside'

Population density:

The colour of each cell indicates the density in that cell as a percentage of the greatest density in any cell of the map.

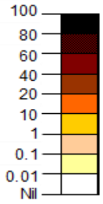

Overall, the tsetse density is within the 'Medium' range for the species.

Area to be baited
