# Supplementary material for: Mathematical modelling and control of African animal trypanosomosis with interacting populations in West Africa—Could biting flies be important in main taining the disease endemicity?
Source: PLoS One. 2020 Nov 20;15(11):e0242435. doi: 10.1371/journal.pone.0242435 (PMC7679153; doi:10.1371/journal.pone.0242435)
Supplement: S4 Fig — (PDF) [file pone.0242435.s004.pdf]

Area to be baited

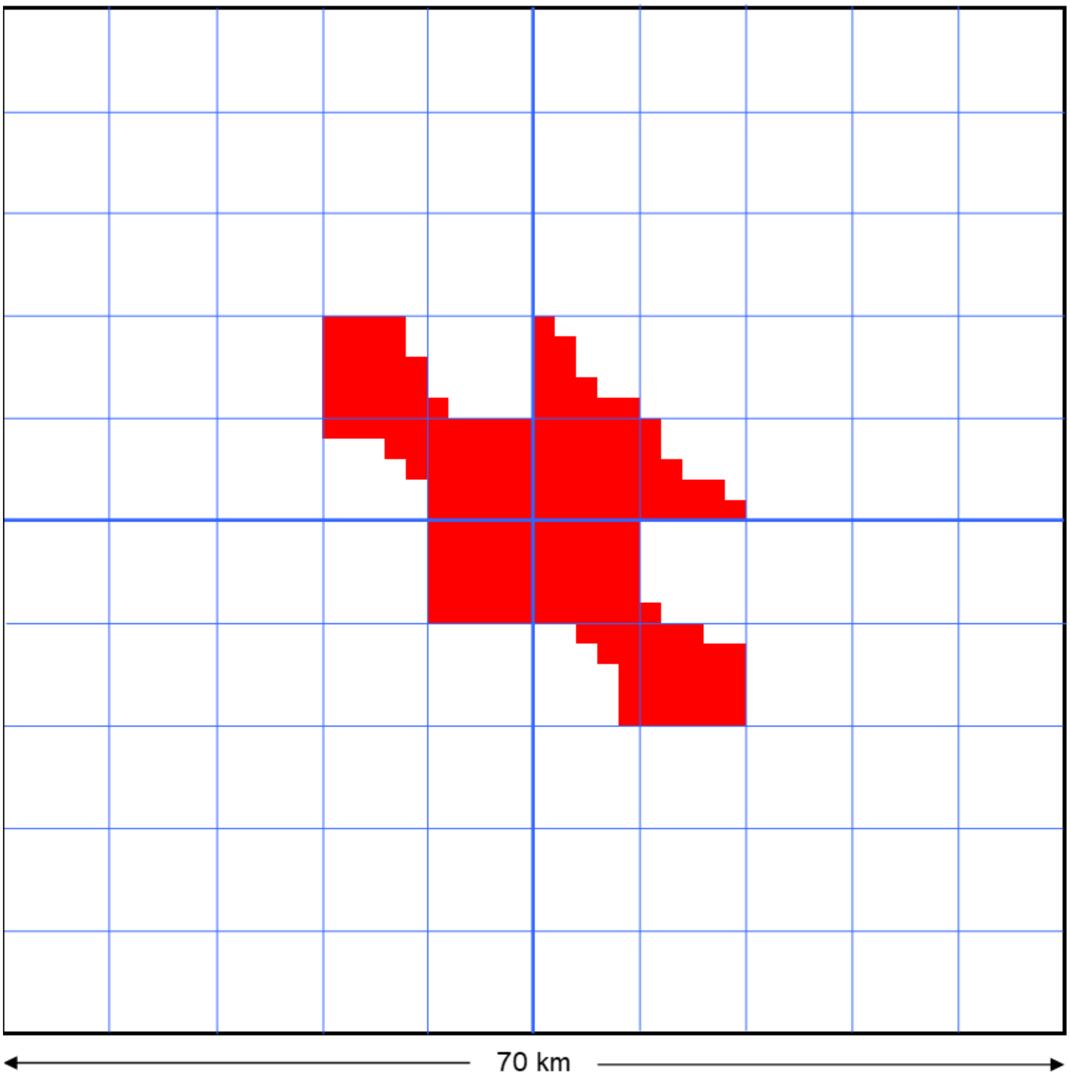

Map consists of 50 x 50 (= 2500) cells, each 1400 x 1400 m square

Area to be baited

Total baited area, km^2: 372

Daily rate of kill to be applied in baited area = 6%

Baits consist of: a) 700 treated cattle
b) 6418 targets (in areas with few cattle)
