# Supplementary material for: Mathematical modelling and control of African animal trypanosomosis with interacting populations in West Africa—Could biting flies be important in main taining the disease endemicity?
Source: PLoS One. 2020 Nov 20;15(11):e0242435. doi: 10.1371/journal.pone.0242435 (PMC7679153; doi:10.1371/journal.pone.0242435)
Supplement: S1 Table — (DOCX) [file pone.0242435.s005.docx]

S1 Table. Elimination cost of tsetse vector in southwest Nigeria.

| Budget | |  |  |  |  |  | |  |
| --- | --- | --- | --- | --- | --- | --- | --- | --- |
|  |  |  |  |  |  |  |  |  |
|  | Item |  |  |  | US$ (thousand) | |  |  |
| 1 | Expendables for baits | | |  |  |  |  |  |
|  | 1.1 | Insecticide-treated cattle | |  |  |  |  |  |
|  |  | 1.1.1 | Insecticide |  | 0.22 |  |  |  |
|  |  | 1.1.2 | Miscellaneous |  | 0.13 |  |  |  |
|  |  |  |  | Sub-total | 0.35 |  |  |  |
|  | 1.2 | Targets | |  |  |  |  |  |
|  |  | 1.2.1 | Cloths |  | 21.31 |  |  |  |
|  |  | 1.2.2 | Supports |  | 0.03 |  |  |  |
|  |  | 1.2.3 | Insecticide |  | 1.76 |  |  |  |
|  |  | 1.2.4 | Miscellaneous |  | 0.06 |  |  |  |
|  |  |  |  | Sub-total | 23.16 |  |  |  |
|  | 1.3 | Monitoring baits | |  |  |  |  |  |
|  |  | 1.3.1 | Traps |  | 2.64 |  |  |  |
|  |  |  |  | Sub-total | 2.64 |  |  |  |
|  |  |  |  |  | Expendables for baits, total | 26.15 |  |  |
| 2 | Transport | |  |  |  |  |  |  |
|  | 2.1 | Mileage (vehicle running costs) | |  |  |  |  |  |
|  |  | 2.1.1 | Light trucks |  | 3.42 |  |  |  |
|  |  | 2.1.2 | Heavy trucks |  | 3.85 |  |  |  |
|  |  |  |  | Sub-total | 7.27 |  |  |  |
|  |  |  |  |  | Transport total | 7.27 |  |  |
| 3 | Facilities | |  |  |  |  |  |  |
|  | 3.1 | Buildings, rent | |  | 400.00 |  |  |  |
|  | 3.2 | Buildings, upkeep | |  | 120.00 |  |  |  |
|  | 3.3 | Water |  |  | 30.00 |  |  |  |
|  | 3.4 | Electricity | |  | 40.00 |  |  |  |
|  |  |  |  |  | Facilities total | 590.00 |  |  |
| 4 | Staff | |  |  |  |  |  |  |
|  | 4.1 | Field staff | |  |  |  |  |  |
|  |  | 4.1.1 | Field manager |  | 1.76 |  |  |  |
|  |  | 4.1.2 | Field assistants |  | 1.52 |  |  |  |
|  |  | 4.1.3 | Labourers |  | 1.13 |  |  |  |
|  |  | 4.1.4 | Field drivers |  | 1.15 |  |  |  |
|  |  |  |  | Sub-total | 5.56 |  |  |  |
|  | 4.2 | Office staff | |  |  |  |  |  |
|  |  | 4.2.1 | HQ manager |  | 0.59 |  |  |  |
|  |  | 4.2.2 | HQ assistants |  | 0.56 |  |  |  |
|  |  | 4.2.3 | HQ drivers |  | 0.77 |  |  |  |
|  |  |  |  | Sub-total | 1.92 |  |  |  |
|  |  |  |  |  | Staff total | 7.48 |  |  |
| 5 | Administration | | |  |  |  |  |  |
|  | 5.1 | Stationery and maps | |  | 25.00 |  |  |  |
|  | 5.2 | Communications | |  | 35.00 |  |  |  |
|  | 5.3 | Computers | |  | 200.00 |  |  |  |
|  | 5.4 | Services | |  | 70.00 |  |  |  |
|  |  |  |  |  | Administration total | 330.00 |  |  |
| 6 | Contingencies | | |  |  |  |  |  |
|  | Total before contingencies | | | 960.90 |  |  |  |  |
|  | Add contingencies at 10% | | |  |  |  |  |  |
|  |  |  |  |  | Contingency total | 96.09 |  |  |
|  | Grand Total | |  |  |  |  |  |  |
|  |  | | |  |  | 1056.99 |  |  |
