# Supplementary material for: Mathematical modelling and control of African animal trypanosomosis with interacting populations in West Africa—Could biting flies be important in main taining the disease endemicity?
Source: PLoS One. 2020 Nov 20;15(11):e0242435. doi: 10.1371/journal.pone.0242435 (PMC7679153; doi:10.1371/journal.pone.0242435)
Supplement: S2 Table — (DOCX) [file pone.0242435.s006.docx]

S2 Table. Elimination strategies for tsetse flies in cattle rearing areas of southwest Nigeria using the tsetse model approach.

| **Details of tactics** | | | | |  |
| --- | --- | --- | --- | --- | --- |
| Insecticide-treated cattle | | | |  |  |
|  | Area with treated cattle, km^2^ | | | | 279 |
|  | Head to be treated | | |  | 700 |
|  | Treatment regime: | | | Surface to cover | Belly and legs |
|  |  | |  | Method of application | Pour-on (RAP) |
|  |  | |  | Average interval of application | 2 weeks |
| **Targets** | | |  |  |  |
|  | | Area with targets, km^2^ | |  | 107 |
|  | | Number to deploy | |  | 6418 |
|  | | Type of target | |  | Blue-black-blue, vertical panels |
|  | | Cloth, finished height x width, cm | | | 100 $\times$ 125 |
|  | | Type of support | |  | Two upright posts + two crossbeams, all wood |
|  | | Is urine used? | |  | No |
|  | | Artificial odour: | | Butanone, mg/h | 100 |
|  | |  |  | Octenol, mg/h | 0.5 |
|  | |  |  | 4-methyl phenol, mg/h | 1 |
|  | |  |  | 3-n-propyl phenol, mg/h | 0.1 |
|  | | Butanone bottle: | | Capacity, ml | 500 |
|  | |  |  | Aperture (hot, medium, cool), mm | 2.5, 3.1, 3.8 |
|  | | Sealed dispenser, Area: Thickness index | | | 0.32 |
| **Survey baits** | |  |  |  |  |
|  | | Traps: |  | Number to deploy | 200 |
|  | |  |  | Type | Nzi |
|  | |  |  | Support | Four wooden struts |
|  | |  |  | Odour dose (x target dose) | 2 |
|  | | Mobile baits: | | Type | No mobile bait to be used |
| **Trypanocide** | |  | |  |  |
|  | | Cost of drug | | per adult cattle | 1.67 |
|  | | Delivery cost | | per adult cattle | 5.56 |
