# Supplementary material for: Mathematical modelling and control of African animal trypanosomosis with interacting populations in West Africa—Could biting flies be important in main taining the disease endemicity?
Source: PLoS One. 2020 Nov 20;15(11):e0242435. doi: 10.1371/journal.pone.0242435 (PMC7679153; doi:10.1371/journal.pone.0242435)
Supplement: Attachment — Submitted filename: rereview.pdf [file pone.0242435.s008.pdf]

Mathematical modelling and control of African animal trypanosomosis with interacting populations in West Africa- could biting flies be important in maintaining the disease endemicity?

Odeniran

Abstract

p.2, l.24 and l.46 trypanosomosis and trypansomiasis used, I'd choose one and stick to it. If you there is a reason for using trypanosomiasis in the keywords, why not use it everywhere?

p.2, l.26 assuming that AAT is regarded as a single disease despite the multiple causative agents, change 'AAT...are a major threat...' to 'AAT...is a major threat...'

p.2, l.33 change '...solving the system of the ordinary...' to '...solving the system of ordinary...'

p.2, l.34 I don't understand how $R_0 < 1$ means you have to control the vectors, doesn't $R_0 < 1$ mean the disease is dying out? I think this part of the abstract is maybe just confused, or lacking some context?

p.2, l.42 change 'individual or together' to 'individually or together'

Introduction

p.3, l.52 'There are other trypanosome species which are also of significant importance to the livestock herd such as *T. evansi* and *T. simiae*.' Are these species being ignored in the modelling? or are all trypanosomes being handled together? in which case I'm not clear why these are differentiated in the text from the 'major' three. Having seen p.4, l.99, I would drop this sentence entirely, being so prominent at the start of the introduction it merely raises questions about something you aren't interested in here.

p.3, l.58 delete 'Generally,' and delete 'annual'

p.3, l.60 I think Tabanidae and Muscidae are families, not genera.

p.3, l.61 'AAT thresholds in various homesteads', I'm not sure about the use of 'thresholds' here, maybe 'levels' would be better.

p.3, l.62 change '...free of tsetse files, where biting flies...' to '...free of tsetse files but where biting flies...'

p.3, l.67 change 'Changes in vector distribution map...' to 'Changes to the vector distribution map...'

p.4, l.78 change '...areas are struggling...' to '...areas is struggling...'

p.4, l.81-83 Do they consider biting flies at all? If not, I'd suggest something more like 'Existing predictive models of African animal trypanosomiasis only consider the biological vectors *Glossina* spp., ignoring the possible importance of mechanical vectors [19,20,21,22]'.

p.4, l.85 some examples of programmes ignoring biting flies and, if there are any, programmes accounting for them would strengthen this. I don't know, but I would guess that no programmes actively seek to eliminate biting flies, but the use of (eg) sprayed insecticides may impact on them as well as the tsetse.

p.4, l.87 And the significance of the variation in daily routines for control is what?

p.4, l.91 the models left knowledge gaps? Or the models suffered as a result of knowledge gaps? I suspect the latter, but I think the sentence implies the former.

p.4, l.99+ I'm not sure about this last paragraph. Partly because I'm not clear that this is what has been done in the study, I don't see how you've evaluated the possibility of the model (I presume you would mean the probability of the model?).

Materials and Methods

p.5, l.112 This is the first reference to bovine trypanosomosis. Why not stick to AAT throughout the manuscript (but making it clear in the intro that your primary interest is in the impact on the the cattle industry)? Do the wildlife and ruminant hosts not get the disease too?

p.5, l.115-6 change '...the cattle input rate of new individuals entering the population. μ_k and τ_k are the...' to '...the rate of new individuals entering the population, while μ_k and τ_k are the...'

p.5, l.117 there are a lot of new terms in here, you should be referencing the table in which all these parameters are defined.

p.6, l.126 do you mean 'domestic livestock cycle' rather than 'domestic cycle'?

p.6, l.126 sentence beginning 'This peculiar...', is there evidence to support this? If so reference it, but maybe in the introduction rather then M&M, alternatively if this is an hypothesis I don't think it should be here. Does it mean that the choice of breeds for use in West Africa results in this siutation? Are the cattle and ruminant breeds used elsewhere in Africa very different? Do they not support trypanosomes? This sentence seems out of place here.

p.6, l.132 and l.141 again there are new undefined parameters, refer to the appropriate table of definitions.

p.7, l.157 change 'climatic dependent' to 'climate-dependent'

p.12, l.200 change '...treated cattle...' to '...treating cattle...'

p.12, l.199 sentence beginning 'Already infected...' I don't fully understand this sentence, but I'm sure there is something wrong with it, may just be the structure of it makes it unclear.

p.12, l.202 what is a trypanocides safety period?

p.12, l.211 The tsetse software is Tsetse Plan? If so, use this name and stick to it. Is this the Tsetse Plan software available from Tsetse.org? Please provide a reference to the software being used, unless it is bespoke, in-house software in which case you should write more about it – here or in the Supplementary material.

p.12, l.211 this heading, with a bracketed alternative heading, reads like you couldn't decide what to call this section. And I'm struggling to think of a suitable heading that covers both parts (a) experimentl and (b) Tsetse Plan evaluation. There is a lot in this section that looks like results and discussion of an experiment, but this is still the M&M section. Here you shoud be saying what you are doing (and maybe just mentioning any result that eg provides a parameter for your model), then have a corresponding section in the Results to present the outputs. I'm not clear that what is done in this section validates your model as stated.

p.13, l.219 I would change 'screened molecularly' to 'screened for trypanosome DNA' as (a) there could be other types of molecular screens and (b) the reader should not have to look up the reference to get that basic information.

p.13, l.237 Sentence 'Therefore, an elimination approach...to control AAT inputs into Tsetse Plan'. Isn't the goal of an elimination approach elimination, rather than provision of inputs to Tsetse Plan? Rewrite.

p.13, l.243 'The population density of the tsetse species was assumed to be medium from the overall report' I assume medium is a setting in the tsetse plan software, but what does it mean as a density? 10 flies per km^2? 100000?

p.13, l.240 I think this paragraph is listing settings used for the Tsetse Plan software. How about a table accompanied by a more succinct statement highlighting those of most interest?

p.13, l251 sentence beginning 'Wildlife is...' does this indicate very low wildlife reservoirs? Only if the grazing areas and wildlife-free areas are more-or-less the same ones. If all the 70km2 are grazing that means there could be a considerable wildlife reservoir in 30km2, couldn't there? Or am I misreading what is there?

p.14-15 I'm struggling to see how the use of Tsetse Plan or the experiment validates your model. The experimental portion provides support for inclusion of biting flies, and provides parameter estimates as inputs to your model, but beyond that I'm confused. And also about how all of this is materials and methods. I'd like to see some kind of schema that explains how your model, the experiment and the Tsetse Plan run(s) link together (what data, parameters etc go from one part to another).

Results

p.17, l.303 doesn't the probability of a fly surviving a feed involve the probability it feeds on an untreated host too? And that sentence need rewriting, eg what is 'feeds off and on treated hosts'?

p.17, l.304 did you predict or assume that flies feed off all cattle at random?

p.17, l.305 change '...systems widely...' to '...systems being widely...'

p.20, (no line given) rewrite the two lines beginning 'In areas where...', I don't get what the bracket is about, there is is a subscript that isn't fully subscripted and the biting flies are abundant (I think).

p.21, l.319 Does the mathematical model confirm the field-data? Didn't the field-data inform the construction of the mathematical model? This seems a bit circular to me.

p.21, l.324? Remove inserted.

p.21, l.325? Remove significantly.
p.21, l.?? change '...poses a risk on...' to '...poses a risk to...'. I don't think I understand the whole of that sentence 'The possibility of continuous treatment...', it is unclear to me what the risk to the herd is from treatment in the presence of infected vectors, please clarify.
p.22, l.327 I assume that there is no requirement in the trypanosome lifecycle to pass through a biological, rather than just mechanical, vector? The lifecycle stages that take place in tsetse can occur just as well elsewhere? Any evidence to support this?
p.23, l.334 change 'It is suffice...' to 'It is sufficient...'
p.23, l.336 is (0.20) an acceptable equation reference in PLOS One? It looks ugly to me, shouldn't it at least be preceded by eq. or equation?
p.24, l.360 change '...that susceptible...' to '...that the susceptible...'
p.24 The two paragraphs that begin 'The graph-based behaviour...', could they be rewritten in such a way as to make differentiate them from one another a bit more. They are very repetitive.
Fig. 3 I can't see a legend so it is unclear what lines refer to what.

p.26, l.397 change 'We observed...' to be 'We estimated...'
p.26, l.399 change 'Meanwhile, if...' to 'However, if…'
p.26, l.400 change '...for longer period, cost...' to '...for a longer period, the cost...'
p.26, l.401 this is the first (and only?) reference to 'fly belt zones' I would recommend avoiding introducing additional terminology at this late point in the paper. You must have described the areas inhabited by flies earlier, reuse that nomenclature.
p.26 The final three sentences on this page, do the all relate to measures to improve the success rate score? It reads as if the first does and then the other two are things that were also done, they should form more of a list. And what does the success rate score of 70% in Tsetse Plan mean?
Discussion
p.27, l.424 spelling 'conditions'
p.27, l.425 spelling 'Tsetse'
p.27, l.427 sentence beginning 'In fact, Rogers...' needs rewriting. I haven't read the reference, but is it the model or the paper that expresses a limitation? And by this do they mean difficulty of including biting flies in the model? The sentence is unclear. I think, and I'm guessing at the meaning a bit, that it something like 'The model of Rogers [19], which was the prototype of subsequent models, omitted biting flies because of the difficulties of including them' or maybe 'including them reliably' if the problem is one of parameterising the models.
p.27, l. 430 change 'helps' to 'help'.
p.27, l.440 sentence (paragraph?) needs rewriting, it implies that complications exist as a result of the model, whereas they are practical considerations which are in place. Either the model should consider them, or perhaps the outcomes of modelling could influence the strategy in the future.
p.28, l.450 Doesn't this also impact tsetse? Or does their strange, for an insect, reproduction make this less likely? I guess so.
p.28, l.464 I think you mean a low rate of infection could persist rather than a slight infection. Or a small population of tsetse through reintroduction/migration result ing on-going infections.
p.28, l.468 '...except cattle are...' implies to me that all cattle are kept in intensive, optimal conditions. Is this right? Or do you mean '...except where cattle are...'?
p.28, l.473 change 'However, it is expected to use artificial baits and targets (ITT) in the other parts where cattle do not visit' to 'However, the use of artificial baits and targets (ITT) is expected in the parts where cattle do not visit'
p.29, l.479 change '...costs...was...' to '...costs...were...'
p.29, l.483 I'm not sure what this sentence means. The tsetse is infected, bites a treated host and transmits to the host before dying. So what is the bit about the tsetse becoming infected through feeding about? And then I don't see how the following sentences carry on from this.
p.30, l.517 I think 'sufficiently eliminated' should be 'eliminated' or 'sufficiently controlled'.

Sensitivity analysis? Variability in parameters?
