# Supplementary material for: Mathematical modelling and control of African animal trypanosomosis with interacting populations in West Africa—Could biting flies be important in main taining the disease endemicity?
Source: PLoS One. 2020 Nov 20;15(11):e0242435. doi: 10.1371/journal.pone.0242435 (PMC7679153; doi:10.1371/journal.pone.0242435)
Supplement: Attachment — Submitted filename: Response to the reviewers.docx [file pone.0242435.s007.docx]

Dear Editor,

Please we have attended to each of the responses from the reviewers below. We hope that this would be satisfactory.

Many thanks

Paul (Corresponding author).

Reviewer #1:

1. The manuscript should be revisited to improve language use. There are a number of confusing non-SVO formated sentences. Just to mention a few, see lines 26-28,39-41, 60-61, 73-76, 79-81, 104-106, 133-153, 180-194, 199-201, 207-210,222-231, 246-247 etc. There is also a great deal of terminology misuse! Just to mention a few; transmitting vectors [may be this means AAT biological and mechanical vectors]. As such some of the section headings e.g. lines 172-173 are equally not understandable

Response: We have corrected the grammatical languages as mentioned by the reviewers.

2. Abstract contains abbreviations that are not written in full first; ITT/ITC

Response: ITT/ITC- means insecticidal treated targets and insecticidal treated cattle. This has been included in the text.

3. The manuscript is incoherent with respect to the mathematical modeling, tsetse plan control and cost simulations and the prevalence studies. It is very hard to rationalize why this paper contains these three sections; these datasets and their implications on AAT transmission dynamics and control are not tied together.

Response: We have improved on the manuscript and also include an aspect in the discussion that reads “Our model showed that apart from tsetse flies which are biological vectors, biting flies remain major drivers in maintaining AAT in West Africa due to their abundance, persistence, resilience to seasonal variations, resistance to insecticides and high reproductive capacity. The prevalence of AAT in vertebrate host and vectoral capacity of biological and mechanical vector flies were important factors in elimination approach. Meanwhile, the southwest Nigeria field model validates our theoretical model because the all the biological and mechanical vectors were observed to harbour trypanosomes, in which our model showed that they have the potential to transmit the pathogen. Hence, eliminating tsetse flies which are K-strategist (species with low reproduction rate) and highly susceptible to insecticides [43, 44], may not necessarily eliminate the biting flies. Hence, more studies are needed on biting flies that could transmit trypanosomes from domestic vertebrates and reservoir hosts due to their interrupted feeding patterns, even when biological vector is absent. Therefore, this model showed that there is a need to concentrate elimination programmes on all the fly vectors with transmission potentials among other control plans like use of trypanocides and institutionalising barriers between domestic and sylvatic cycles”.

4. Unless it is a peculiar AAT eco-epidemiological situation in Nigeria for which you need to provide references, sheep and goats suffer from clinical AAT. It is therefore surprising that they are classified together with wild animals and treated as mere reservoirs of AAT in this manuscript. Small ruminants should therefore be modeled as part of the AAT domestic cycle.

Response: Small ruminants develop clinical AAT of heavy challenge of trypanosomes. In endemic region, small ruminants need to be treated to be in recovered class. Although, in the case of persistent, those in recovered class could return to susceptible class if exposed to trypanosome infection. Meanwhile, at subclinical level of trypanosome infection, they are thought to maintain the disease in the domestic cycle, serving as reservoirs for Trypanosoma species. These peculiar AAT eco-epidemiological situation in West Africa could largely be attributed to breed selection over the years. They rightly fit in SIR model, which has now been included in the work.

5. The expression for R_0 given on page 6 seems not to be coming from the proposed model. A citation for this expression should be provided. There is also no link between this expression and the proposed model.

Response: We have decided to develop an expression for R_0 in the model now.

6. Some equations on page 7 like (0.4) and (0.5) are missing the derivative sign

Response: The derivative sign has now been included as $\frac{{dR}_{k}}{dt}$ and $\frac{{dS}_{t}}{dt}$ in equation 0.4 and 0.5

7. The model development indicates that tsetse flies get infected when feeding on infected cattle, wildlife and small ruminants (page 6, line 126). This is not reflected in the model. What I can see in the model is that tsetse flies get infection from infected cattle only. Basically, the model needs to be revised. Adding a model diagram will also be helpful.

Response: Thank you for the observation. Additional models including infection of tsetse flies and biting flies from infected small ruminants and wildlife has been included (Equation 0.12 – 0.16). Moreover, we have included a schematic diagram for easy explanation of the model).

8. There is no derivation given for expressions of the basic reproduction numbers R_01 and R_02. On page 12 after equation (0.12), the authors indicate that the next generation matrix technique earlier described was used. The earlier description of this technique cannot be seen. This article can be used for this technique. Please refer to; *Van den Driessche, P., & Watmough, J. (2002). Reproduction numbers and sub-threshold endemic equilibria for compartmental models of disease transmission. Math. Biosci., 180, 29–48.

Response: We have shown the derivation and included the techniques of Diekmann et al., 1990 and Van den Driessche, P., and Watmough, J. (2002). These two authors have been cited, and the technique was applied to our model.

9. One page 5, line 114, the cattle variables are defined, the same should be done for the variables of other populations considered in the model for easy follow up. All model variables need to be defined.

Response: We have defined other variables e.g. small ruminants and wildlife properly.

10. Results for local stability of the disease-free equilibrium and endemic equilibrium are not shown. The following articles can assist in doing both local and global stability analysis of the disease-free and endemic equilibrium. Please refer to;
a) *Chavez, C. C., Feng, Z., & Huang, W. (2002). On the computation of R0 and its role on global stability. Mathematical Approaches for Emerging and Re-Emerging Infection Diseases: An Introduction. The IMA Volumes in Mathematics and Its Applications, 125, 31-65.
b) *McCluskey C. C.(2006), Lyapunov functions for tuberculosis models with fast and slow progression. Mathematical Biosciences and Engineering, 3(4), 603C614
c) *Korobeinikov A. (2004), Lyapunov functions and global properties for SEIR and SEIS epidemic models. Mathematical Medicine & Biology: A Journal of the IMA. 21(2)(2004).
d) Other similar publications on local and global stability analysis can be downloaded online.

Local stability of an equilibrium would imply existence of that situation only for a short time (depending on certain circumstances or conditions). Whereas, global stability would imply existence of a situation regardless of any condition. For example, 1. If an endemic equilibrium is globally stable, it would imply that in the long run, the disease prevails (i.e. it is not cured- it becomes endemic- it is not eliminated) 2. If a disease-free equilibrium is globally stable it would imply that the disease finally dies out. 3. If a disease-free equilibrium is locally stable, it would imply that the disease would be eliminated (provided that certain conditions are met or for a short time depending on certain conditions). Mathematically, when global stability is performed, there is no need to perform local stability. However, in our-build-up on the global stability analyses of disease-free and endemic equilibrium, we have stated the local stability of disease-free and endemic equilibrium theorem, which implies the stability. We have also cited the suggested authors.

11. For AAT, recovered animals become susceptible again. Additionally, an animal infected with T. b. brucei, for example, is susceptible to other trypanosome species. This has not been taken care of in the mathematical model

Response: Thank you for your response. The model illustrates how the recovered class could become susceptible again. We have indicated it properly in the diagram (Fig 1). If an animal is infected with T. b. brucei and also susceptible to other trypanosome species, the recovery route is very essential. For instance, if infected animal is treated with trypanocide, the drug protects it from getting infected to other species within the protective period. We have explained this properly in the work.

12. There is apparent lack of methods description with regard to both prevalence and tsetse plan [control options and their relative costs].

Response: The methods on prevalence studies and control plans (which incorporated relative costs) have been properly explained.

13. Presentation of results should be improved by only presenting the model outputs. All models and their parameterization should be presented in methods section. As well, the last section of results; lines 298-312 reads like methods and not results.

Response: All models and parameterization have now been moved to the method section. The last section of the results on budgeting and compiling final report has been improved upon and now reads like results.

14. Insecticidal resistance is not known for tsetse; Tsetse are R-strategists [first paragraph of results section.

Response: The insecticide resistance was meant for biting flies which could pose continuous challenge of trypanosome infections. Tsetse are K-strategists, which have been properly explained in the last paragraph of the discussion.

15. Please quantify the effectiveness of ITC/ITT eluded in lines 239-240 and elsewhere in this manuscript.

Response: We have explained the differences in effectiveness of ITC/ITT in the manuscript. It has been recast thus “Due to pastoralism and nomadism management system widely practised in West Africa, ITC is very effective because livestock farmers move their animals around for grazing. For ITT, beyond the target animals were considered, in which the vector fly populations of adjacent areas were also targeted. This is an effective method in elimination strategy, it also targets flies within and without target areas. It is most effective in areas where zero-grazing is practised. The use of ITC was more effective than ITT which affected the feeding tsetse and other biting flies, largely because of management practices”.

16. There are generic statements e.g. “behavior of tsetse”, behavior of cattle, “the magnitude of the exposed cattle ….in lines 274-293 that render almost all that section impossible to comprehend.

Response: The word behaviour is attributed to the graph-based representation, while magnitude expresses the size number of the object in mathematics. We have explained this at the first mention for the readers.

17. This MS treats tsetse and biting flies as equal vectors [mechanical or biological] without due regard to the type of AAT. T. brucei and T. congolense are biologically transmitted by tsetse and T. vivax by both mechanical and biological transmission! I don’t see this taken care of at model parameter description, setup and parameterization. As a result, numerical results presented in lines 274-293 don’t seem to make lots of epidemiological sense to me. Please also check that discussion lines 322-331, 340-359 make epidemiological sense. There are lots of inaccuracies presented in these lines.

Response: We have improved the discussion. We earlier stated that contaminated biting flies only transmit *T. vivax*.

18. Table 2; check that “whole fly” and “troublesome” make sense in the context of this MS

Response: We have removed the whole fly and corrected troublesome to trypanosome.

19. For Tables S1 and S2: Once you have provided a detailed methods section for these outputs, the results presented in these tables should give the reader an indication of the cost per animal per year [parasite control costs] or cost for vector control /km2 for a specified time needed for suppression or elimination. These costs should be discussed citing an existing body of knowledge about costs. The reader’s attention should be drawn to the methods of cost estimation deployed in tsetse plan to those implemented elsewhere before such costs can be compared.

Response: This has been included in the method section, “The cost analyses is technique dependent. In this study two viable techniques in West Africa considered in the model were trapping, ITC and ITT. In cases of animal movement where sufficient livestock are present for ITC, insecticides are often applied by spraying (RAP has been cost effective) or pour on. The cost of traps is relatively high (not necessarily the cost of acquiring the traps), because of the cost of manpower required for deployment which is dependent on density. Notably, the targeted vector flies were Glossina species and biting flies, which means that combination of techniques were required”.

The result section has also been updated with “We observed the cost for elimination of an isolated study area with Palpalis group of tsetse flies and biting flies to cost between 599 - 1875 US$ / km^2^. However, to maintain barriers against reinvasion for the next five years, the cost could add between 20 - 50% to the original cost. Meanwhile, if barriers are extended to larger areas for longer period, cost would increase further. The treatment of cattle in protected areas would be between 4 - 12 times in fly belt zones, depending on the severity of fly challenge from Glossina species and biting flies (S1 Table and S2 Table). The price of trypanocides was estimated at US$ 1.67 for >150 kg adult dose and delivery cost at US$ 5.56, bringing the cost per dose at US$ 0.036 / kg and thus US$ 28.9 per cattle / annum if administered quarterly. The cost could increase to US$ 115.6 / cattle / annum if trypanocides are administered monthly. Hence, if targeted areas are well-protected (traps, ITC, ITT), the trypanocide treatment would only be quarterly”.

In the discussion, the work of Shaw, 2009 has been referenced and compared with the findings on the cost analysis.

Reviewer #2: I found it difficult to engage with the study, and I believe this is down in part to the structure of the paper and some missing information.

I will give a small number of examples.
In the Materials and Methods, the section on the Nigeria case study (starting on page 8) contains a lot of information which discusses previous work, without being clear what the study you are reporting on does in this area (either materials or methods). What is the tsetse software? and tsetse plan? How do they work? Are they the implementations of your model? Or are values derived from the model being fed into them to generate outputs? Does using this software validate the model in some way? Or extend it's outputs (eg into the economics or operational planning).

Response: We have greatly improved the materials and methods, results and discussion sections. The prevalence of AAT in vertebrate host and vectoral capacity of biological and mechanical vector flies were important factors in elimination approach. Meanwhile, the southwest Nigeria field model validates our theoretical model because the all the biological and mechanical vectors were observed to harbour trypanosomes, in which our model showed that they have the potential to transmit the pathogen. The values from southwest Nigeria were entered in out Tsetse Plan software which automatically generates the scenario. Some of these field data were also important in our mathematical model. The whole concept was to validate our model with the field data to develop the best control approach.

I have spent a lot of time going backwards and forwards trying to find bits of information, or where things were first described. Possibly my own fault for printing the paper rather than viewing on screen. However, “To obtain R0 for the the model (2.1)-(2.11), the next generation matrix technique earlier described were utilised” appear on page 12 but the only previous reference to next generation matrix technique is in the abstract. So if it is described it isn't clearly labelled as such.

Response: The derivation of the next generation matrix has been improved upon and clearly stated. Many thanks

The model equations are referenced in numerous places as (2.1)-(2.11), but in my copy of the manuscript the equations appear to be labelled (0.1)-(0.11).

Response: Many thanks for the observation. We have corrected the labelling now.

There are a large number of grammatical and spelling mistakes, which I am unwilling to spend time on now given that the paper requires a substantial restructuring to separate material into its appropriate section and clarify how the different aspects of the study tie together. On next review, I would spend more time on the language.

Response: Many thanks for offering to help with the language. We will appreciate that. We also gave it to native English speakers for review to minimise the spelling mistakes.
